# A Rare Case of Pembrolizumab-Induced Reactivation of Hepatitis B

**DOI:** 10.1155/2018/5985131

**Published:** 2018-10-17

**Authors:** Anita Pandey, Susan Ezemenari, Maksim Liaukovich, Ivan Richard, Avezbakiyev Boris

**Affiliations:** Department of Hematology and Oncology, Brookdale University Hospital, Medical Center, Brooklyn, USA

## Abstract

Hepatitis B virus (HBV) infection is common across the world, especially in Asia, Africa, Southern Europe, and Latin America. The association of HBV infection in patients suffering from different oncological conditions is well established. Many cases of HBV reactivation have been reported in patients on immunosuppressive chemotherapy and in patients undergoing hematopoietic bone marrow transplantations. Only one case has been reported so far of HBV reactivation in a patient treated with programmed cell death receptor 1 (PD-1) checkpoint inhibitors in the setting of HIV status. We report a case of a 51-year-old male, former smoker, diagnosed with stage IV poorly differentiated adenocarcinoma of the lung, and started on pembrolizumab, who developed reactivation of chronic hepatitis requiring antiviral therapy.

## 1. Introduction

Reactivation of HBV is defined as a more than 10-fold rise in HBV DNA, detection of HBV DNA in a patient that was previously undetectable, or when reverse seroconversion occurs (i.e., when an HBsAg-negative/anti-HBc positive patient becomes HBsAg positive) [1]. HBV reactivation is a well-known complication of immunosuppressive therapy which can present as fulminant hepatitis, liver failure, and even death [2, 3]. Approximately 240 million people are positive for HBsAg worldwide [4]. The incidence rate of HBV infection reactivation during immunosuppressive chemotherapy is 14–72% [5]. There is strong evidence that reactivation of HBV can be prevented by screening and prophylaxis in patients on immunosuppressive therapy [6, 7]. Most reported cases of HBV reactivation have involved hematological malignancies treated with chemotherapy [8]. However, corresponding data for patients suffering from adenocarcinoma of the lung and treated with PD-1/PDL-1 inhibitors is not known except for one case report of an HBV flare in a patient with HIV and stage III non-small cell lung cancer (NSCLC) treated with nivolumab [9]. We report a case of HBV reactivation in a patient diagnosed with adenocarcinoma of the lung, treated with pembrolizumab. After initiating antiviral treatment, our patient had complete resolution of his acute hepatitis and continues his immunotherapy treatment till date.

## 2. Case Presentation

A 51-year-old male, former smoker and former alcoholic, presented to our emergency department with a few weeks' history of headache associated with left-sided weakness, without fever, seizures, nausea, or visual impairment. A neurological exam was significant for left hemiparesis. CT head was remarkable for multiple isodense and hypodense lesions in the frontal lobes, right parietal lobes, and cerebellum suspicious for metastatic lesions. CT chest was significant for a nodular density in the medial right upper lobe and right hilar lymph node. Biopsy of the lung mass and the hilar lymph node revealed poorly differentiated adenocarcinoma. Immunohistochemistry was positive for TTF-1 (thyroid transcription factor-1), Napsin, and PDL-1 expression of >95% PDL-1. NGS (next-generation sequencing) was negative for EGFR mutation. Treatment for metastatic adenocarcinoma of the lung was initiated based on these findings.

After the completion of whole brain radiation, the patient was started on pembrolizumab as the first-line therapy. His baseline complete blood count (CBC), comprehensive metabolic panel (CMP), and thyroid stimulating hormone (TSH) were normal. In the setting of the normal liver function test and absence of symptoms, hepatitis panel was not indicated and not performed at baseline. Following the first cycle of pembrolizumab, a rise in ALT (Alanine aminotransferase) to 528 U/L (normal range: 9–52 U/L) and AST (Aspartate aminotransferase) to 342 U/L (normal range: 14–36 U/L) was noted. Consequently, pembrolizumab was held, and over the next few days, ALT peaked to 994 U/L and AST to 670 U/L. Total bilirubin and alkaline phosphatase were normal. Treatment for probable autoimmune hepatitis was started with high-dose steroids tapered over 3 weeks. The patient's liver enzymes remained elevated in spite of the steroids. Hepatitis workup was sent which revealed HBsAg positive, anti-HBsAb negative, and total anti-HBc positive while IgM anti-Hbc was negative, HbeAg was nonreactive, and HbeAb was reactive which was consistent with chronic hepatitis B. In addition to the serological markers, presence of newly elevated transaminases and HBV DNA RT-PCR (real-time polymerase chain reaction) of >8.23 log was consistent with HBV reactivation. Other tests including ANA, smooth muscle antibody, and ferritin were within normal limits.

After the initiation of tenofovir treatment, liver enzymes started to trend downward. Pembrolizumab was reintroduced and continued without any further significant events. Liver enzymes returned to normal range within a 10-week period, and HBV DNA was undetectable.

## 3. Discussion

The HBV virus is a double-stranded DNA virus which induces host immune response in hepatocytes via MCH II-CD4+ helper T cells and MCH I-CD8+ cytotoxic T cells [10]. The release of IFN-gamma, TNF-alpha, and chemokines downregulates viral replication in liver cells. When the cytotoxic T cell response cannot clear the virus completely, chronic infection persists. HBV exhibits immune tolerance during chronic infection by depleting virus-specific T cells [10]. Intrahepatic covalently closed circular DNA (cccDNA) in chronic carriers is vital for HBV replication while on conventional chemotherapy [11]. These chemoagents suppress the immune system thus encouraging viral replication and increasing viral DNA levels. Additionally, peroxisome proliferator-activated receptor-gamma coactivator-1 alpha (PGC-1 alpha), a key regulator of cellular processes like gluconeogenesis and fatty acid oxidation in hepatocytes, is linked to HBV induction [11, 12]. It has been postulated that high-dose steroids stimulate glucocorticoid-responsive elements in the HBV genome resulting in an increase in transcriptional activity [13], whereas monoclonal antibodies like rituximab and alemtuzumab cause prolonged B and T cell depletion, increasing the risk of HBV reactivation-linked fatal hepatitis along with other severe infections [14]. The pathophysiology behind immunotherapy-induced reactivation may not be parallel with conventional chemotherapies and immunosuppressant-induced reactivation as it is a promoter of T cells which is responsible for the clearance of the virus from the host. Therefore, further molecular studies are needed to explore and clarify the mechanism.

Pembrolizumab is a humanized monoclonal antibody which inhibits PD-1 activity by binding to the PD-1 receptor in T cells, therefore reversing T cell immune suppression and promoting T cell proliferation and cytotoxicity [15, 16]. With the extended FDA approval of pembrolizumab for all solid tumors with high microsatellite instability (MSI-H), indications for the drug have broadened beyond NSLC (non-small cell lung cancer) [17]. With the increased use of pembrolizumab, the incidence of cases similar to that which we report will probably rise. Though the prevalence of HBV will vary in different populations, we strongly feel that screening for chronic HBV infection by checking HBsAg and anti-HBc should be a standard practice prior to starting immunotherapy. However, two vital questions require further evaluation. First, should the entire patient population that has screened positive for chronic hepatitis be placed on antiviral therapy prior to starting immunotherapy? Second, what should be the duration of antiviral therapy?

Patients who had prior infection with HBV virus (positive for HBsAg or anti-HBc antibody) and are receiving immunosuppressive therapy for malignancy, autoimmune disease, or solid organ/hematopoietic stem cell transplant are at high risk for reactivation and are therefore indicated for antiviral prophylaxis [18, 19]. In most cases, antiviral therapy was held after 6 months of completion of immunosuppression [14].

In conclusion, immunotherapy can lead to HBV reactivation and systematic screening for chronic hepatitis before initiating therapy is justifiable. Our patient has stage IV lung adenocarcinoma and will probably remain on immunotherapy until the progression of his disease or until the evidence of toxicity. His viral load is undetectable, and his treatment regimen continues to include tenofovir therapy.

## 4. Our Recommendations

Screen all patients receiving pembrolizumab for evidence of chronic hepatitis B infection by testing for HBsAg and anti-HBcAb. Concurrent administration of antiviral prophylaxis (i.e., tenofovir or entecavir) in patients with moderate to high risk for HBV reactivation should be considered.
